# Efficacy and safety of adjunctive clobazam in Chinese patients with drug-resistant epilepsy: a single-center real-world study

**DOI:** 10.3389/fneur.2026.1757055

**Published:** 2026-03-26

**Authors:** Yaqian Zhang, Xianyue Liu, Zhengwei Su, Xiaowei Xu, Yingfang She, Shuda Chen, Liemin Zhou

**Affiliations:** 1 Department of Neurology, The Seventh Affiliated Hospital, Sun Yat-sen University, Shenzhen, Guangdong, China; 2 Department of Neurology, Henan Provincial People’s Hospital, Zhengzhou University People’s Hospital, Zhengzhou, Henan, China

**Keywords:** adjunctive therapy, clobazam, drug-resistant epilepsy, efficacy, safety, seizure

## Abstract

**Objective:**

This study evaluated the efficacy and safety of adjunctive clobazam (CLB) in Chinese patients with drug-resistant epilepsy (DRE).

**Methods:**

We retrospectively included 121 DRE patients receiving adjunctive CLB at the Epilepsy Center of the Seventh Affiliated Hospital of Sun Yat-sen University (Jan 2023-May 2025). Patients were followed at 1, 3, 6, 9, and 12 months or longer. Data collected included epilepsy etiology, seizure type, frequency, treatment duration, dosage, and adverse events. Response was defined as ≥50% reduction in seizure frequency from baseline.

**Results:**

A total of 121 patients were included (age at medication use 32.4 ± 13.5 years; mean three prior failed antiseizure medications; mean follow-up 12 months). Seizure types were focal (18.2%), generalized (7.4%), and both (74.4%). At first follow-up, 51.2% achieved ≥50% seizure reduction and 39.7% were seizure freedom; at last follow-up, 85.9% responded and 51.6% were seizure freedom. Response rates increased over time (all *p* < 0.001). Adults had higher response (*p* = 0.046) and seizure freedom rates (*p* = 0.041) than children. Concomitant oxcarbazepine (OXC) was associated with increased seizure freedom (*p* = 0.03), whereas valproate (VPA) was associated with low response rate (*p* = 0.013) but not with seizure freedom. Age at medication (OR = 1.032, *p* = 0.035), CLB dosage (OR = 1.093, *p* = 0.004), and OXC co-therapy (OR = 2.311, *p* = 0.04) were independent predictors of seizure freedom. Most common adverse events were somnolence/mental fatigue (9.9%) and hypersomnia (5%).

**Conclusion:**

Adjunctive CLB significantly reduced seizures in DRE with sustained efficacy. Higher dosage, longer treatment duration, and concomitant OXC use were associated with improved outcomes. CLB showed favorable efficacy, wide applicability, and acceptable safety.

## 1 Introduction

Epilepsy is a common chronic neurological disorder affecting approximately 60 million people worldwide, characterized by recurrent seizures that substantially impair quality of life and social functioning (1). Although antiseizure medications (ASMs) effectively control seizures in the majority of patients, nearly one-third remain refractory to treatment with two or more adequately dosed ASMs and ultimately develop drug-resistant epilepsy (DRE) (2–4). For this population, monotherapy is often insufficient, making polytherapy a necessary strategy. The selection of adjunctive agents must balance efficacy, tolerability, and drug–drug interactions, while also accounting for epilepsy syndrome, age, and comorbid conditions (5).

Clobazam (CLB), a 1,5-benzodiazepine with both antiepileptic and anxiolytic properties, represents a promising option for adjunctive therapy (6). Unlike conventional 1, 4-benzodiazepines, CLB selectively targets the α2 subunit of the GABA_A receptor, resulting in weaker sedative effects, improved tolerability, and reduced risk of tolerance with long-term use (7). These pharmacological advantages make it particularly suitable for patients with DRE. Since its approval by the U. S. Food and Drug Administration (FDA) in 2011 as adjunctive therapy for seizures associated with Lennox–Gastaut syndrome (LGS) in patients ≥2 years old, CLB has been increasingly studied. Clinical evidence supports its efficacy in pediatric-onset refractory epilepsies, including LGS (8, 9), Dravet syndrome (DS) (10), and epilepsy with myoclonic-atonic seizures (EMAS) (11). Moreover, smaller studies have suggested potential benefits of CLB as monotherapy or adjunctive therapy in adults with refractory epilepsy (12, 13). Nevertheless, systematic data on its long-term effectiveness, safety, treatment patterns, and adverse effects across diverse epilepsy syndromes and larger patient cohorts remain limited.

In September 2022, domestically manufactured clobazam (10 mg × 28 tablets, produced by Yichang Humanwell) received formal approval from the National Medical Products Administration (NMPA) of China, providing Chinese patients with DRE a legal and more affordable treatment option, and creating an opportunity to evaluate its therapeutic potential across broader patient populations. This retrospective study, conducted at a tertiary epilepsy center, aimed to assess the effectiveness and safety of adjunctive CLB in patients with DRE. By contributing real-world evidence from China, our findings are expected to inform clinical practice and further enrich the global evidence base for CLB in DRE.

## 2 Methods

### 2.1 Patient selection

**Figure 1:**
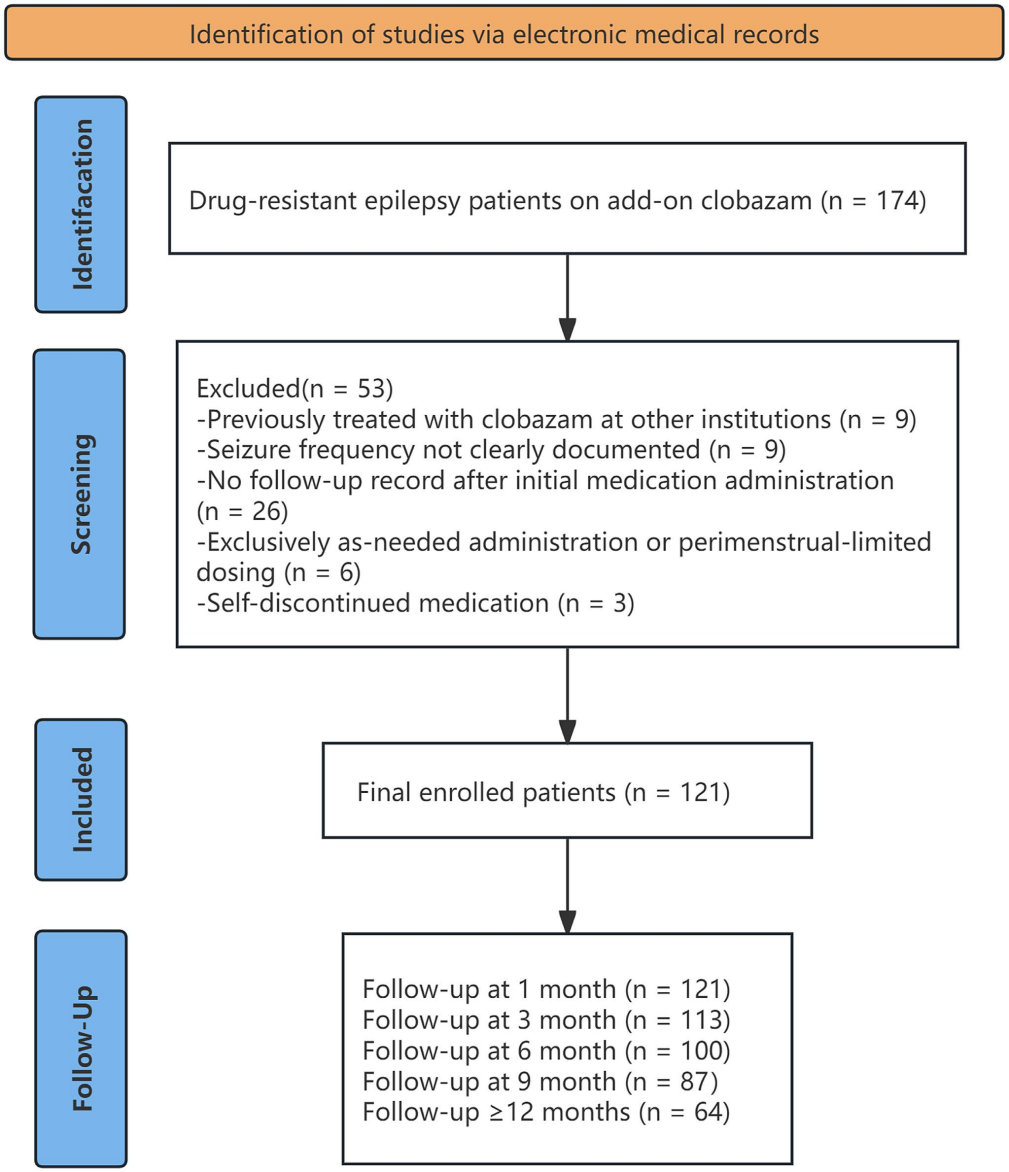
Flowchart of patient selection and follow-up among patients with drug-resistant epilepsy receiving adjunctive clobazam.

We retrospectively identified patients with DRE (4) (defined as the failure of adequate trials of two appropriately chosen and tolerated antiseizure medication regimens to achieve sustained seizure freedom),who received adjunctive CLB at the Epilepsy Center, Seventh Affiliated Hospital, Sun Yat-sen University, between January 2023 and May 2025 (*n* = 174). Patients were excluded if they had prior CLB exposure before referral, incomplete seizure frequency records, no follow-up after initiation, intermittent or menstrual-related use only, or self-discontinuation. A total of 121 patients met inclusion criteria (Figure 1).

### 2.2 Data collection

Demographic and clinical variables were collected, including age, sex, epilepsy duration, etiology, seizure type and frequency, comorbidities, medical and surgical history, neuroimaging findings, and concomitant ASMs. Follow-up visits were scheduled at 1, 3, 6, 9, and 12 months after CLB initiation and thereafter.

### 2.3 Outcome measures

Outcomes included seizure frequency, CLB dose adjustments, and adverse events. Seizure frequency was determined at each visit based on parent-reported seizure diaries and summarized as the average number of seizures per month. CLB dose adjustments and adverse events were recorded at each follow-up visit. All clinical information, including medication details, dose changes, and reported adverse events, was documented by treating physicians in the hospital electronic medical record system. Adverse events were captured through a retrospective review of outpatient clinic notes, hospitalization records, and follow-up documentation in the medical record system. For each follow-up time point within the same patient, treatment response was defined as seizure freedom or a ≥ 50% reduction in seizure frequency compared with baseline. Non-response was defined as a < 50% reduction in seizure frequency compared with baseline.

### 2.4 Statistical analyses

Data were analyzed using SPSS 24.0 (IBM, United States). Continuous variables were summarized as mean ± SD or median (IQR) and compared with t test or Mann–Whitney U test, as appropriate. Categorical variables were expressed as n (%) and compared with ***χ***^**2**^ or Fisher’s exact test; for multiple comparisons of categorical variables, Bonferroni correction was applied to adjust the significance level. Predictors of seizure freedom were examined with binary logistic regression. CLB retention was estimated using Kaplan–Meier analysis, and survival curves were plotted with R (version 4.3.1).

### 2.5 Ethics approval

This study follows the principles of the Declaration of Helsinki. Approval was granted by the Ethics Committee of Seventh Affiliated Hospital of Sun Yat-sen University.

## 3 Results

### 3.1 Patient characteristics and demographics

A total of 121 patients with DRE who received CLB as adjunctive therapy for ≥1 month were included. The mean age of onset was 23.4 ± 14.6 years, with a nearly equal male-to-female ratio. The median duration of epilepsy was 8 years (IQR: 3–15), and patients had previously failed an average of 3 ASMs (IQR 2–3). Most patients experienced both focal and generalized seizures (74.4%), while 18.2% had focal seizures and 7.4% had generalized seizures. Neuroimaging revealed structural lesions in 61.2% of patients. Structural etiology was the most common cause (54.5%), followed by genetic (13.2%), immune (11.6%), and unknown etiologies (20.7%). Past medical history included febrile seizures (5.0%), encephalitis (4.1%), traumatic brain injury (4.1%), and cerebral ischemia/hypoxia (3.3%). Twenty-three patients (19.0%) had undergone surgical treatment, most commonly epileptogenic focus resection (12.4%). The most frequent comorbidities were intellectual disability (12.4%), mood disorders (9.9%), and cognitive impairment (6.6%). The median initial CLB dose was 10 mg/day (IQR: 5–10), which increased to 15 mg/day (IQR: 10–20) at the last follow-up. The median follow-up duration was 12 months (IQR: 6.5–15.5). The concomitant use of CLB in this cohort primarily involved valproate (49.6%), oxcarbazepine (48.8%), levetiracetam (43%), and lamotrigine (28.1%) (Table 1).

**Table 1:** Baseline demographic and clinical characteristics of the patients.

Clinical characteristics	Value
Age at medication initiation, mean ± SD	32.4 ± 13.5
Age of onset, mean ± SD	23.4 ± 14.6
Sex	
Male, *n* (%)	59 (48.8)
Female, *n* (%)	62 (51.2)
Duration of epilepsy (year), median (IQR)	8(3–15)
Seizure type	
Focal, *n* (%)	22 (18.2)
Generalized, *n* (%)	9 (7.4)
Both, *n* (%)	90 (74.4)
MR/PET-CT imaging	
Non-lesional, *n* (%)	31 (25.6)
Lesional, *n* (%)	74 (61.2)
Not evaluated, *n* (%)	16 (13.2)
Etiology	
Structural, *n* (%)	66 (54.5)
Immune, *n* (%)	14 (11.6)
Genetic, *n* (%)	16 (13.2)
Unknown etiology, *n* (%)	25 (20.7)
Past medical history	
Febrile seizures, *n* (%)	6 (5.0)
Encephalitis, *n* (%)	5 (4.1)
Autoimmune diseases, *n* (%)	2 (1.7)
Cerebral ischemia and hypoxia, *n* (%)	4 (3.3)
Traumatic brain injury, *n* (%)	5 (4.1)
Cerebrovascular accident, *n* (%)	2 (1.7)
Surgical history	
Epileptogenic focus surgical resection, *n* (%)	15 (12.4)
SEEG-guided radiofrequency thermocoagulation, *n* (%)	3 (2.5)
Vagus nerve stimulation, *n* (%)	5 (4.1)
Comorbidities	
Autism spectrum disorder, *n* (%)	1 (0.8)
Intellectual disability, *n* (%)	15 (12.4)
Mood disorders (anxiety/depression), *n* (%)	12 (9.9)
Cognitive impairment, *n* (%)	8 (6.6)
Sleep disorders, *n* (%)	3 (2.5)
Attention-deficit/hyperactivity disorder, *n* (%)	2 (1.7)
Chronic headache, *n* (%)	2 (1.7)
Spastic cerebral palsy, *n* (%)	1 (0.8)
Number of ASMs before CLB treatment, median(IQR)	3 (2–3)
Initial CLB dose (mg/d), median (IQR)	10 (5–10)
CLB dosage at final follow-up (mg/d), median (IQR)	15 (10–20)
Follow-up duration (month), median (IQR)	12 (6.5–15.5)
Frequently used regimens with CLB	
Valproate (VPA), *n* (%)	60 (49.6)
Oxcarbazepine (OXC), *n* (%)	59 (48.8)
Levetiracetam (LEV), *n* (%)	52 (43.0)
Lamotrigine (LTG), *n* (%)	34 (28.1)
Perampanel (PER), *n* (%)	26 (21.5)
Topiramate (LPM), *n* (%)	14 (11.6)
Lacosamide (LCM), *n* (%)	13 (10.7)
Carbamazepine (CBZ), *n* (%)	9 (7.4)
Zonisamide (ZNS), *n* (%)	2 (1.7)
Phenobarbital (PB), *n* (%)	2 (1.7)

ASMs, antiseizure medications; CLB, clobazam.

### 3.2 Association between CLB efficacy and clinical characteristics

#### 3.2.1 Different time points of administration

At the 1-month follow-up, 51.2% of patients responded to CLB treatment, with response rates gradually increasing over time, reaching 85.9% at ≥12 months. Statistical analysis revealed significant differences in response rates (*p* < 0.001) across different follow-up time points. After Bonferroni correction, response rates at 6, 9, and 12 months were all significantly higher than at 1 month (all *p* < 0.001), and response rates at 9 and 12 months were also significantly higher than at 3 months (*p* = 0.002 and *p* < 0.001, respectively). In contrast, differences in seizure freedom rates (*p* = 0.515) across follow-up time points were not statistically significant. Nevertheless, the proportion of seizure freedom patients increased from 39.7% at 1 month to 51.6% at ≥12 months, while the proportion of non-responders decreased progressively over time. The median dose of CLB increased from 10 mg/day (IQR: 10–10) at 1 month to 15 mg/day (IQR: 10–20) at 6 months and remained stable thereafter. These findings suggest that the efficacy of adjunctive CLB improves progressively with prolonged treatment and dose optimization (Table 2).

**Table 2:** Efficacy of adjunctive clobazam therapy in epilepsy patients at different time points.

Changes in seizure frequency and CLB dosage	Follow-up time point(s)				
	1 month (*n* = 121)	3 months (*n* = 113)	6 months (*n* = 100)	9 months (*n* = 87)	≥12 months (*n* = 64)
Responders (*n*, %)	62 (51.2%)^*^	68 (60.2%)^†^	75 (75.0%)^*^	70 (80.5%)^*,†^	55 (85.9%)^*,†^
Seizure freedom (*n*, %)	48 (39.7%)	44 (39.0%)	40 (40.0%)	37 (42.5%)	33 (51.6%)
≥50% reduction (*n*, %)	14 (11.6%)	24 (21.2%)	35 (35.0%)	33 (38.0%)	22 (34.3%)
Non-responders (*n*, %)	59 (48.8%)	45 (39.8%)	25 (25.0%)	17 (19.5%)	9 (14.1%)
<50% reduction (*n*, %)	15 (12.4%)	16 (14.2%)	9 (9.0%)	6 (6.9%)	4 (6.3%)
No improvement (*n*, %)	44 (36.4%)	29 (25.7%)	16 (16.0%)	11 (12.6%)	5 (7.8%)
CLB dosage(mg/d)	10 (10–10)	10 (10–20)	15 (10–20)	15 (10–20)	15 (10–20)

*6 months vs. 1 month, *p* < 0.001; 9 months vs. 1 month, *p* < 0.001; 12 months vs. 1 month, *p* < 0.001. ^†^9 months vs. 3 months, *p =* 0.002; 12 months vs. 3 months, *p* < 0.001.

#### 3.2.2 Different etiologies

Analysis by epilepsy etiology showed that patients with structural and autoimmune causes had the highest response rates, at 80.3 and 78.6%, respectively. Patients with unknown etiology exhibited a response rate of 72.0%, whereas those with genetic etiology had a relatively lower response rate of 56.3%. Regarding seizure freedom, the highest proportion was observed in the structural etiology group (48.5%), followed by the unknown etiology group (44.0%) and the autoimmune etiology group (35.7%), with the lowest rate in the genetic etiology group (18.8%). Overall, adjunctive CLB demonstrated efficacy across all etiological groups, with the structural etiology group achieving the highest response and seizure freedom rates and the genetic etiology group the lowest. However, the differences in response rates and seizure freedom rates among the groups did not reach statistical significance (*p* > 0.05) (Supplementary Table S1).

#### 3.2.3 Age at medication initiation stratified analysis

In the age at medication use stratified analysis, the overall response rate was significantly higher in adults (≥18 years) at 81.3%, compared with 65.2% in the pediatric group (<18 years) (*p* = 0.046). Similarly, the seizure freedom rate was significantly greater in adults, reaching 49.3% *vs.* 30.4% in the pediatric group (*p* = 0.041). These findings indicate that adjunctive CLB is more effective in adult patients with epilepsy than in pediatric patients (Table 3).

**Table 3:** Efficacy of adjunctive clobazam therapy in adult and pediatric epilepsy patients.

Changes in seizure frequency and CLB dosage	Age group	
	Pediatric (<18 years) (*n* = 46)	Adult (≥18 years) (*n* = 75)
Responders (*n*, %)	30 (65.2%)^*^	61 (81.3%)^*^
Seizure freedom (*n*, %)	14 (30.4%)^†^	37 (49.3%)^†^
≥50% reduction (*n*, %)	16 (34.8%)	24 (32.0%)
Non-responders (*n*, %)	16 (34.8%)	14 (18.6%)
<50% reduction (*n*, %)	5 (10.9%)	2 (2.6%)
No improvement (*n*, %)	11 (23.9%)	12 (16.0%)
CLB dosage(mg/d)	20 (12.5–25)	15 (10–20)

*Adult vs. Pediatric, *p* = 0.046. ^†^Adult vs. Pediatric, *p* = 0.041.

#### 3.2.4 Different disease durations

In the analysis stratified by epilepsy duration, patients with a disease course of 0–10 years exhibited the highest response rate (76.5%), followed by those with a duration of 10–20 years (75.0%), while patients with a duration >20 years had a relatively lower response rate (66.7%). Overall, response rates showed a declining trend with longer disease duration, although the differences were not statistically significant. With respect to seizure freedom, the highest proportion was observed in patients with disease duration >20 years (50.0%), followed by those with 0–10 years (41.9%) and 10–20 years (39.3%). Taken together, adjunctive CLB demonstrated therapeutic benefits across all duration groups, but there were no statistically significant differences in response rates or seizure freedom rates among the groups(*p* > 0.05) (Supplementary Table S2).

#### 3.2.5 Different seizure types

Based on seizure type stratification, the highest response rate was observed in patients with combined seizure types (76.6%), followed by those with focal seizures (72.7%) and generalized seizures (66.6%). With respect to seizure freedom, the focal seizure group achieved the highest rate (45.4%), followed by the combined seizure group (42.2%), while the generalized seizure group showed a relatively lower rate (33.3%). Overall, adjunctive CLB therapy demonstrated favorable efficacy across different seizure types; however, no statistically significant differences in response rates were observed among the groups (*p* > 0.05) (Supplementary Table S3).

#### 3.2.6 CLB in combination with different ASMs

Among patients receiving CLB in combination with different ASMs, efficacy differed across treatment regimens. Compared with the CLB + Others group, the response rate was significantly lower in the CLB + VPA + Others group (55.6% *vs.* 84.6%, *p* = 0.013) (Table 4). The seizure freedom rate was significantly higher in the CLB + OXC + Others group than in the CLB + Others group (54.3% *vs.* 26.9%, *p* = 0.03). For ≥50% seizure reduction, the CLB + Others group (57.7%) demonstrated higher rates than both the CLB + OXC + Others group (28.6%, *p* = 0.025) and the CLB + VPA + Others group (22.3%, *p* = 0.006).

**Table 4:** Efficacy of adjunctive clobazam therapy in epilepsy patients with receiving different ASMs.

Parameter	ASMs regimen			
	CLB + Others (*n* = 26)	CLB + OXC + Others (*n* = 35)	CLB+VPA + Others(*n* = 36)	CLB + OXC + VPA + Others (*n* = 24)
Etiology				
Structural (*n*, %)	15 (57.7%)	23 (65.7%)	13 (36.2%)	15 (62.5%)
Immune (*n*, %)	5 (19.3%)	3 (8.6%)	3 (8.3%)	3 (12.5%)
Genetic (*n*, %)	3 (11.5%)	0 (0%)	12 (33.3%)	1 (4.2%)
Unknown (*n*, %)	3 (11.5%)	9 (25.7%)	8 (22.2%)	5 (20.8%)
Seizure type				
Focal (*n*, %)	5 (19.2%)	7 (20%)	5 (13.9%)	5 (20.8%)
Generalized (*n*, %)	2 (7.7%)	0 (0%)	7 (19.4%)	0 (0%)
Both (*n*, %)	19 (73.1%)	28 (80%)	24 (66.7%)	19 (79.2%)
Responders (*n*, %)	22 (84.6%)	29 (82.9%)	20 (55.6%)^*^	20 (83.3%)
Seizure freedom (*n*, %)	7 (26.9%)	19 (54.3%)^†^	12 (33.3%)	13 (54.2%)
≥50% reduction (*n*, %)	15 (57.7%)^☨^	10 (28.6%)	8 (22.3%)	7 (29.2%)
Non-responders (*n*, %)	4 (15.4%)	6 (17.1%)	16 (44.4%)	4 (16.7%)
<50% reduction (*n*, %)	0 (0%)	2 (5.7%)	4 (11.1%)	1 (4.2%)
No improvement (*n*, %)	4 (15.4%)	4 (11.4%)	12 (33.3%)	3 (12.5%)
CLB dosage(mg/d)	15 (10–25)	15 (10–20)	20 (10–25)	10 (10–20)

^†^CLB + OXC + Others vs CLB + Others, *p* = 0.03. *CLB + VPA+Others *vs.* CLB+Others, *p* = 0.013. ☨CLB + OXC+Others vs CLB + Others, *p* = 0.025; CLB + VPA + Others vs. CLB + Others, *p* = 0.006.

Baseline characteristics differed among groups. The proportion of genetic etiology was higher in the CLB + VPA + Others group, whereas no genetic cases were observed in the CLB + OXC + Others group. No significant differences were identified in structural, immune, or unknown etiologies (*p* > 0.05). Seizure type distribution also differed across groups, with a higher proportion of generalized seizures in the CLB + VPA + Others group, while focal and both seizure types were comparable (*p* > 0.05). Accordingly, subgroup analyses stratified by etiology were conducted.

Among patients with structural epilepsy (Table 5), the response rates were 87.0% in the CLB + OXC + Others group and 69.2% in the CLB + VPA + Others group, with no significant differences compared with CLB + Others (p > 0.05). However, the seizure freedom rate was significantly higher in the CLB + OXC + Others group than in CLB + Others (60.9% *vs.* 26.7%, *p* = 0.044). In patients with non-genetic epilepsy (Table 6), the seizure freedom rate was also higher in the CLB + OXC + Others group compared with CLB + Others (54.3% *vs.* 26.1%, *p* = 0.05). Additionally, the ≥50% seizure reduction rate in the CLB + Others group (56.5%) was significantly higher than that in both the CLB + VPA + Others group (20.8%, *p* = 0.018) and the CLB + OXC + Others group (28.6%, *p* = 0.041).

**Table 5:** Efficacy of adjunctive clobazam therapy in patients with structural epilepsy receiving different ASMs.

Changes in seizure frequency and CLB dosage	ASMs regimen			
	CLB + Others (*n* = 15)	CLB+OXC + Others (*n* = 23)	CLB+VPA + Others (*n* = 13)	CLB + OXC + VPA + Others (*n* = 15)
Responders (*n*, %)	12 (80%)	20 (87.0%)	9 (69.2%)	12 (80%)
Seizure freedom (*n*, %)	4 (26.7%)	14 (60.9%)^*^	5 (38.4%)	9 (60%)
≥50% reduction (*n*, %)	8 (53.3%)	6 (26.1%)	4 (30.8%)	3 (20%)
Non-responders (*n*, %)	3 (20%)	3 (13%)	4 (30.8%)	3 (20%)
<50% reduction (*n*, %)	0 (0%)	1 (4.3%)	1 (7.7%)	1 (6.7%)
No improvement (*n*, %)	3 (20%)	2 (8.7%)	3 (23.1%)	2 (13.3%)
CLB dosage(mg/d)	20 (10–30)	15 (10–20)	20 (15–25)	10 (10–20)

*CLB + OXC + Others vs. CLB + Others, *p* = 0.044.

**Table 6:** Efficacy of adjunctive clobazam therapy in patients with non-genetic epilepsy receiving different ASMs.

Changes in seizure frequency and CLB dosage	ASMs regimen			
	CLB + Others (*n* = 23)	CLB + OXC + Others (*n* = 35)	CLB+VPA + Others (*n* = 24)	CLB + OXC + VPA + Others (*n* = 23)
Responders (*n*, %)	19 (82.6%)	29 (82.9%)	15 (62.5%)	19 (82.6%)
Seizure freedom (*n*, %)	6 (26.1%)	19 (54.3%)*	10 (41.7%)	13 (56.5%)
≥50% reduction (*n*, %)	13 (56.5%)^†^	10 (28.6%)	5 (20.8%)	6 (26.1%)
Non-responders (*n*, %)	4 (17.4%)	6 (17.1%)	9 (37.5%)	4 (17.4%)
<50% reduction (*n*, %)	0 (0%)	2 (5.7%)	3 (12.5%)	1 (4.3%)
No improvement (*n*, %)	4 (17.4%)	4 (11.4%)	6 (25.0%)	3 (13.1%)
CLB dosage(mg/d)	15 (10–25)	15 (10–20)	20 (10–25)	10 (10–20)

^*^CLB+OXC+Others vs. CLB+Others, *p* = 0.05. ^†^CLB+OXC+Others vs. CLB+Others, *p* = 0.041; CLB+VPA+Others vs. CLB+Others, *p* = 0.018.

For the CLB + LEV + Others group, 73.0% of patients achieved response, including 38.5% who achieved seizure freedom, with no significant difference compared with CLB + Others (Supplementary Table S4). Similarly, in the CLB + LTG + Others group, 73.5% of patients responded, including 44.1% who achieved seizure freedom, also without significant difference compared with CLB + Others (Supplementary Table S5).

### 3.3 Predictors of seizure freedom

Univariate analysis indicated that age at medication initiation (OR = 1.031, 95%CI: 1.002–1.061, *p* = 0.033), CLB dose (OR = 1.101, 95%CI: 1.039–1.166, *p* = 0.001), and combination therapy with OXC (OR = 2.682, 95%CI: 1.274–5.646, *p* = 0.009) were significantly associated with seizure freedom. Multivariate analysis further demonstrated that age at medication initiation (Adjusted OR = 1.032, 95%CI: 1.002–1.064, *p* = 0.035), CLB dose (Adjusted OR = 1.093, 95%CI: 1.029–1.161, *p* = 0.004), and combination therapy with OXC (Adjusted OR = 2.311, 95%CI: 1.040–5.132, *p* = 0.04) remained independent predictors (Table 7).

**Table 7:** Binary logistic regression analysis of predictors of seizure freedom in patients.

Risk factors	seizure free (*n* = 51)	seizure present (*n* = 70)	OR (95%CI)	*p*	Adjusted OR (95%CI)	Adjusted *p*
Sex						
Male, *n*	30 (58.8%)	29 (41.4%)	/	/		
Female, *n*	21 (42.1%)	41 (58.6%)	0.495 (0.238–1.030)	0.06		
Age at medication initiation (year), mean ± SD	33.1 ± 13.0	17.1 ± 12.2	1.031 (1.002–1.061)	0.033	1.032 (1.002–1.064)	0.035
Duration of epilepsy (year), mean ± SD	9.6 ± 7.2	9.5 ± 7.8	1.002 (0.955–1.052)	0.928		
Seizure type						
Focal, *n*	10 (19.6%)	12 (17.1%)	/	/		
Generalized, *n*	3 (5.9%)	6 (8.6%)	0.600 (0.119–3.032)	0.537		
Both, *n*	38 (74.5%)	52 (74.3%)	0.877 (0.343–2.240)	0.784		
Comorbidities, *n*	16 (31.4%)	21 (30.0%)	1.067 (0.488–2.331)	0.871		
MR/PET-CT imaging						
Non-Lesional, *n*	10 (19.6%)	21 (30.0%)	/	/		
Lesional, *n*	35 (68.6%)	39 (55.7%)	1.885 (0.781–4.546)	0.158		
Not evaluated, *n*	6 (11.8%)	10 (14.3%)	1.260 (0.357–4.449)	0.720		
Etiology						
Structural, *n*	32 (62.7%)	34(48.6%)	/	/		
Immune, *n*	5 (9.8%)	9 (12.9%)	0.590 (0.179–1.950)	0.387		
Genetic, *n*	3 (5.9%)	13 (18.6%)	0.245 (0.064–0.941)	0.041		
Unknown etiology, *n*	11 (21.6%)	14 (20.0%)	0.835 (0.331–2.106)	0.702		
Positive medical history, *n*	10 (19.6%)	14 (20.0%)	0.954 (0.394–2.414)	0.957		
Surgical history, *n*	7 (13.7%)	14 (20.0%)	0.636 (0.237–1.712)	0.371		
Follow-up time(month), median (IQR)	12 (9–16)	9.5 (6–15)	1.033 (0.977–1.092)	0.254		
CLB dose(mg/d), median (IQR)	20 (10–25)	10 (10–20)	1.101 (1.039–1.166)	0.001	1.093 (1.029–1.161)	0.004
Number of ASMs, median (IQR)	3 (3–4)	3 (3–4)	0.817 (0.508–1.314)	0.405		
Combination with VPA, *n*	25 (49.0%)	35 (50.0%)	0.962 (0.467–1.979)	0.915		
Combination with OXC, *n*	32 (62.7%)	27 (45.6%)	2.682 (1.274–5.646)	0.009	2.311 (1.040–5.132)	0.04

### 3.4 Adverse events

During adjunctive CLB treatment, adverse events were generally infrequent. The most common events were somnolence and mental fatigue (9.9%, 12/121), followed by hypersomnia (5.0%, 6/121). Limb weakness and decreased appetite each occurred in 4.1% of patients (5/121). Other events, including periumbilical pain, falls, irritability, respiratory depression, memory impairment, hand tremor, headache, blurred vision, and menorrhagia, were reported in ≤1% of patients (1/121 each). In subgroup analysis, somnolence, mental fatigue, or hypersomnia occurred in 11 patients (18.3%) in the CLB + VPA group and 7 patients (11.4%) in the CLB + Others group, with no statistically significant difference (*p* > 0.05). Among patients with adverse events, the dose of CLB was 20 (10–20) mg/day. One patient experienced respiratory depression, which resolved after dose reduction from 20 to 10 mg/day and another experienced intolerable hypersomnia at 10 mg/day, which resolved after reduction to 5 mg/day. The remaining patients tolerated their current dose without adjustment. No patients discontinued CLB due to adverse events. Overall, CLB was well tolerated, with most adverse events being mild to moderate and predominantly affecting the central nervous system (Table 8).

**Table 8:** Adverse events associated with adjunctive clobazam therapy.

Adverse events	*n* (%)
Somnolence and mental fatigue	12 (9.9%)
Hypersomnia	6 (5%)
Limb weakness	5 (4.1%)
Poor appetite	5 (4.1%)
Periumbilical pain	1 (0.8%)
Fall(s)	1 (0.8%)
Irritability	1 (0.8%)
Sensation of respiratory depression	1 (0.8%)
Memory impairment	1 (0.8%)
Hand tremor	1 (0.8%)
Headache	1 (0.8%)
Blurred vision	1 (0.8%)
Menorrhagia	1 (0.8%)

### 3.5 Drug retention rate

**Figure 2:**
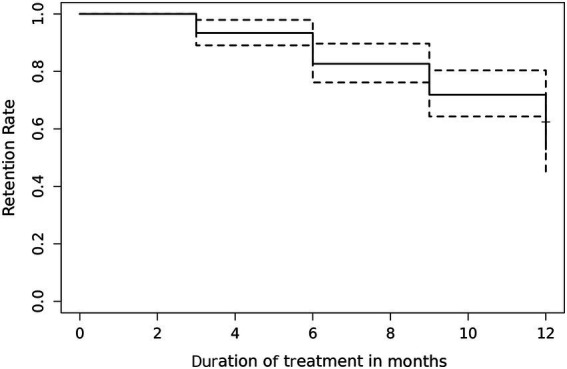
Kaplan–Meier analysis for estimated retention rate of patients on clobazam (*n* = 121).

During follow-up, 23 patients discontinued CLB, with the main reasons being a change in treatment regimen (*n* = 18) and economic reasons (*n* = 5). To further evaluate long-term adherence, Kaplan–Meier analysis was performed among 121 patients receiving adjunctive CLB. Early retention after 3 months was 93% and gradually declined to 53% at 12 months. This decline reflects both treatment discontinuation among some patients and variable observation periods. Patients who had not yet reached the 12-month time point were censored in the analysis, allowing estimation of retention rates despite incomplete follow-up. These findings indicate favorable tolerability and adherence, supporting the clinical applicability and long-term therapeutic value of CLB as an adjunctive treatment in patients with epilepsy (Figure 2).

## 4 Discussion

This study, based on real-world retrospective cohort data from a single tertiary epilepsy center in China, evaluated the efficacy and safety of CLB in patients with DRE. The results demonstrated that adjunctive treatment with CLB significantly reduced seizure frequency in DRE patients, with its efficacy progressively enhanced over time through extended follow-up and dose optimization. At the last follow-up (≥12 months), 85.9% of patients achieved ≥50% reduction in seizure frequency compared with baseline, and 51.6% attained complete seizure control, with no serious adverse events observed. These findings suggest that CLB is an effective and well-tolerated therapeutic option for patients with DRE.

CLB, a long-acting benzodiazepine synthesized in 1966, has been widely used for both anxiolytic and antiepileptic purposes. Although initially approved for the treatment of LGS, its off-label use in both pediatric and adult patients with DRE has increased in recent years (14). Previous randomized controlled trials and retrospective studies have reported that more than 50% of patients with DRE achieved ≥50% seizure reduction after CLB add-on therapy (15–18), and approximately 40%–70% attained seizure freedom during long-term follow-up beyond 1 year (12, 18, 19). However, these studies have generally been limited by small sample sizes and modest evidence quality. Our findings further support the effectiveness of adjunctive CLB therapy. At the first follow-up, 51.2% of patients achieved ≥50% seizure reduction and 39.7% achieved complete seizure freedom. By the last follow-up (median: 12 months), the response rate and seizure freedom rate had increased to 85.9 and 51.6%, respectively, in parallel with higher CLB dosages. These results suggest that the therapeutic efficacy of CLB in patients with DRE improves progressively with longer treatment duration and gradual dose escalation. We therefore propose that sustained treatment and careful dose optimization are critical for achieving optimal outcomes in clinical practice.

Subgroup analyses stratified by etiology revealed that patients with structural causes exhibited the highest response and seizure freedom rates, whereas those with genetic causes showed relatively lower rates. Although differences between etiological groups did not reach statistical significance, the overall trend suggests that CLB exerts efficacy across diverse DRE etiologies. Further stratification by seizure type indicated that patients with focal seizures had relatively higher response and seizure freedom rates, consistent with previous reports (12, 20, 21). When stratified by disease duration, a declining trend in response rate was observed with longer disease course, although differences were not statistically significant. Collectively, these findings indicate that CLB demonstrates considerable efficacy across different etiologies, seizure types, and disease durations, supporting its potential utility across a broad spectrum of epilepsy patients.

Our study identified age at CLB initiation and CLB dosage as important predictors of seizure freedom. Adult patients exhibited higher response and seizure freedom rates compared with pediatric patients, and older age at CLB initiation was independently associated with a greater likelihood of achieving seizure freedom in regression analysis. This association may reflect intrinsic differences between adult and pediatric populations rather than a true timing effect. Pediatric patients in our cohort had a higher prevalence of genetic etiologies, which are often associated with more refractory seizure patterns. In addition, developmental pharmacokinetic variability, challenges in dose optimization, and adherence issues in younger patients may have contributed to the observed lower response and seizure freedom rates in this group. Further prospective studies are warranted to clarify the independent impact of treatment timing on CLB response. Moreover, higher CLB doses were positively associated with seizure freedom rates, suggesting that gradual dose escalation within tolerable limits may improve outcomes. These findings are consistent with the dose–response relationship previously observed in patients with LGS (22, 23). Taken together, our findings suggest that adult patients, particularly those receiving sodium-channel blockers such as oxcarbazepine, may derive substantial benefit from adjunctive CLB therapy, whereas pediatric patients with genetic epilepsies may experience comparatively lower rates of complete seizure freedom while still achieving meaningful clinical improvement. This observation may help clinicians tailor expectations and adjunctive treatment strategies for different patient subgroups.

In this study, the most commonly co-administered ASMs included VPA, OXC, LEV and LTG. The results showed that concomitant VPA use was associated with a lower response rate, whereas OXC co-administration was associated with an increased seizure freedom rate. Interestingly, patients receiving CLB + VPA had a lower response rate than those in the CLB + Others group. This discrepancy is likely due to the higher proportion of patients with genetic epilepsy in the CLB + VPA group, who are typically more drug-resistant and have more complex disease courses, thereby limiting treatment efficacy. Pharmacologically, VPA primarily exerts its antiepileptic effects by enhancing GABAergic inhibition and modulating voltage-gated sodium and T-type calcium channels (24). The overlap in GABAergic mechanisms may limit additional benefits from the combination, although VPA’s modulation of ion channels could provide some complementary effects. Furthermore, adverse events such as somnolence, mental fatigue, and hypersomnia were more frequent in the CLB + VPA group, potentially constraining further dose escalation. Previous pharmacokinetic studies have shown that VPA can accelerate N-CLB clearance (10.5%) (25), but with minimal impact on overall plasma concentrations and pharmacological effects (26). Notably, in subgroup analyses excluding patients with genetic etiologies, the response rate of CLB + VPA was comparable to that of CLB + Others, suggesting that the reduced efficacy was primarily driven by underlying genetic factors. In contrast, the higher seizure freedom rate observed in the CLB + OXC group may reflect complementary mechanisms of action, with CLB enhancing GABAergic inhibition and OXC blocking voltage-gated sodium channels (5). These complementary mechanisms could contribute to additive or potentially synergistic antiseizure effects (27). Adjunctive treatment with OXC was identified as an independent predictor of seizure freedom, particularly among patients with structural or non-genetic epilepsy. Collectively, these findings indicate that CLB efficacy is influenced by both patient-specific factors, such as etiology and seizure type, and the pharmacological properties of concomitant ASMs. Individualized combination strategies that consider both mechanistic complementarity and patient-specific disease characteristics may optimize therapeutic outcomes. Further prospective, randomized, double-blind studies are warranted to validate these observations and to elucidate potential pharmacokinetic and pharmacodynamic interactions.

Regarding safety, this study indicates that CLB is generally well tolerated. The most common adverse events were somnolence and fatigue (9.9%), which were mostly mild to moderate in severity. These effects are likely related to CLB’s action as a 1, 5-benzodiazepine on the central nervous system, enhancing GABA-mediated inhibitory neurotransmission and reducing neuronal excitability, which, while controlling seizures, may also induce mild sedation or fatigue (28). Other adverse events, including limb weakness, decreased appetite, periumbilical pain, irritability, headache, and cognitive impairment, occurred in ≤5% of patients, with no serious or life-threatening reactions observed, indicating overall good tolerability. Kaplan–Meier analysis showed that early adherence to CLB was nearly 100%, and approximately 53% at 12 months, suggesting favorable tolerability and adherence during long-term use.

Nevertheless, this study has several limitations. First, its retrospective single-center design may introduce selection bias and limit the generalizability of the findings. Second, the relatively small sample size in some subgroups reduced the statistical power of stratified analyses. In addition, long-term neurocognitive outcomes, quality of life measures, and pharmacokinetic data were not systematically collected. Future multicenter, prospective studies with larger cohorts are needed to validate these findings and to further explore efficacy, safety, and the impact of concomitant ASMs across different patient populations.

## 5 Conclusion

In summary, adjunctive CLB significantly reduces seizure frequency in patients with DRE, with efficacy enhanced by dose escalation and longer treatment duration. CLB has demonstrated favorable effectiveness, broad applicability, and acceptable safety across diverse patient populations. Future multicenter, prospective studies with larger sample sizes are warranted to further validate these findings.

## Data Availability

The original contributions presented in the study are included in the article/Supplementary material, further inquiries can be directed to the corresponding authors.
