# Supplementary material for: Efficacy and safety of adjunctive clobazam in Chinese patients with drug-resistant epilepsy: a single-center real-world study
Source: Front Neurol. 2026 Mar 26;17:1757055. doi: 10.3389/fneur.2026.1757055 (PMC13061665; doi:10.3389/fneur.2026.1757055)
Supplement: Supplementary file 1 [file Supplementary_file_1.docx]

Supplementary Table S1. Efficacy of adjunctive clobazam therapy by epilepsy etiology

| **Changes in seizure frequency and CLB dosage** | **Etiology classification** | | | |
| --- | --- | --- | --- | --- |
|  | **Structural**  **(n=66)** | **Immune**  **(n=14)** | **Genetic**  **(N=16)** | **Unknown**  **(n=25)** |
| **Responders (n, %)**  seizure freedom (n, %)  ≥50% reduction (n, %) | 53(80.3%)  32(48.5%)  21(31.8%) | 11(78.6%)  5(35.7%)  6(42.9%) | 9(56.3%)  3(18.8%)  6(37.5%) | 18(72.0%)  11(44.0%)  7(28.0%) |
| **Non-responders (n, %)**  <50% reduction (n, %)  No improvement (n, %) | 13(19.7%)  3(4.6%)  10(15.1%) | 3(21.4%)  2(14.3%)  1(7.1%) | 7(43.7%)  1(6.2%)  6(37.5%) | 7(28.0%)  1(4.0%)  6(24.0%) |
| **CLB dosage(mg/d)** | 17.5(10-20) | 17.5(15-20) | 15(10-20) | 20(10-20) |

There was no statistically significant difference between the groups.

Supplementary Table S2. Efficacy of adjunctive clobazam therapy in epilepsy patients with different disease durations

| **Changes in seizure frequency and CLB dosage** | **Disease duration** | | |
| --- | --- | --- | --- |
|  | **0<y≤10**  **(n=81)** | **10<y≤20**  **(n=28)** | **y>20**  **(n=12)** |
| **Responders (n, %)**  seizure freedom (n, %)  ≥50% reduction (n, %) | 62(76.5%)  34(41.9%)  28(34.6%) | 21(75.0%)  11(39.3%)  10(35.7%) | 8(66.7%)  6(50.0%)  2(16.7%) |
| **Non-responders (n, %)**  <50% reduction (n, %)  No improvement (n, %)  **CLB dosage(mg/d)** | 19(23.5%)  6(7.4%)  13(16.1%)  20(10-20) | 7(25.0%)  1(3.6%)  6(21.4%)  20(20-30) | 4(33.3%)  0(0.0%)  4(33.3%)  20(15-20) |

There was no statistically significant difference between the groups.

Supplementary Table S3. Efficacy of adjunctive clobazam therapy by seizure type in epilepsy patients

| **Changes in seizure frequency and CLB dosage** | **Seizure type** | | |
| --- | --- | --- | --- |
|  | **Focal**  **(n=22)** | **Generalized**  **(n=9)** | **Both**  **(n=90)** |
| **Responders (n, %)**  seizure freedom (n, %)  ≥50% reduction (n, %) | 16(72.7%)  10(45.4%)  6(27.3%) | 6(66.6%)  3(33.3%)  3(33.3%) | 69(76.6%)  38(42.2%)  31(34.4%) |
| **Non-responders (n, %)**  <50% reduction (n, %)  No improvement (n, %)  **CLB dosage(mg/d)** | 6(27.3%)  1(4.6%)  5(22.7%)  15(10-20) | 3(33.3%)  0(0%)  3(33.3%)  12.5(10-20) | 21(23.3%)  6(6.7%)  15(16.7%)  20(15-25) |

There was no statistically significant difference between the groups.

Supplementary Table S4. Efficacy of adjunctive clobazam therapy in epilepsy patients receiving different ASMs

| **Changes in seizure frequency and CLB dosage** | **ASMs regimen** | |
| --- | --- | --- |
|  | **CLB+Others**  **(n=69)** | **CLB+LEV+Others**  **(n=52)** |
| **Responders (n, %)**  seizure freedom (n, %)  ≥50% reduction (n, %) | 53(76.8%)  32(46.4%)  21(30.4%) | 38(73.0%)  19(36.5%)  19(36.5%) |
| **Non-responders (n, %)**  <50% reduction (n, %)  No improvement (n, %)  **CLB dosage(mg/d)** | 16(23.2%)  3(4.3%)  13(18.8%)  20(15-20) | 14(27.0%)  4(7.7%)  10(19.3%)  15(10-20) |

There was no statistically significant difference between the groups.

Supplementary Table S5. Efficacy of adjunctive clobazam therapy in epilepsy patients receiving different ASMs

| **Changes in seizure frequency and CLB dosage** | **ASMs regimen** | |
| --- | --- | --- |
|  | **CLB+Others**  **(n=87)** | **CLB+LTG+Others**  **(n=34)** |
| **Responders (n, %)**  seizure freedom (n, %)  ≥50% reduction (n, %) | 66(75.9%)  36(41.4%)  30(34.5%) | 25(73.5%)  15(44.1%)  10(29.4%) |
| **Non-responders (n, %)**  <50% reduction (n, %)  No improvement (n, %)  **CLB dosage(mg/d)** | 21(24.1%)  7(8.0%)  14(16.1%)  20(12.5-25) | 9(26.5%)  0(0.0%)  9(26.5%)  20(10-20) |

There was no statistically significant difference between the groups.
